# Portal Vein Thrombosis in the Setting of Newly Diagnosed Cushing’s Syndrome

**DOI:** 10.1177/2324709617703672

**Published:** 2017-04-10

**Authors:** Alexandria D. McDow, Anju Gurung, Rama Poola, Carmel Fratianni, Marc Garfinkel, Michael G. Jakoby

**Affiliations:** 1 Southern Illinois University, Springfield, IL, USA

**Keywords:** portal vein thrombosis, hypercoagulability, hypercortisolism, Cushing’s syndrome

## Abstract

The hallmark manifestations of Cushing’s syndrome (CS) are well known, but hypercoagulability is perhaps least recognized. Patients with CS are at increased risk of both spontaneous and postoperative thromboembolism, with the significant majority of events occurring in the lower extremity and pulmonary venous circulations. We present a case of portal vein thrombosis (PVT) occurring in the setting of newly diagnosed CS due to a left adrenal adenoma. Factor VIII activity was approximately 2.5-fold elevated, a known mechanism by which hypercortisolemia predisposes to venous thrombosis. Acute abdominal pain and fever responded well to unfractionated heparin and parenteral antibiotics, and CS was eventually cured by left adrenalectomy. No thromboembolic events have occurred since surgery. PVT is uncommon and usually occurs as a complication of primary or secondary hepatobiliary malignancies and cirrhosis. To the best of our knowledge, this is just the second reported case of PVT due to CS and the first published in the English language literature.

## Introduction

Cushing’s syndrome (CS) is caused by chronic exposure to excess glucocorticoid, with endogenous CS due to elevated levels of cortisol. Endogenous CS may be caused by corticotrope adenomas in the pituitary (Cushing’s disease), ectopic corticotropin (ACTH) production by peripheral tumors of neuroendocrine origin (eg, small cell carcinoma), or adrenal adenomas and carcinomas that autonomously overproduce cortisol. The adverse effects of excess cortisol are wide ranging and include centripetal obesity, sarcopenia, insulin resistance, hypertension, immunosuppression, and accelerated bone loss. Endogenous CS patients have a 2-fold higher rate of mortality than unaffected individuals that persists even after successful treatment.^1^

An important but less commonly recognized complication of hypercortisolism is hypercoagulability and venous thromboembolism (VTE). CS patients have an increased risk of both spontaneous and postoperative VTE. In a systematic review of 15 patient series, the incidence of spontaneous or postoperative VTE for CS patients was approximately 10-fold higher than the estimated incidence in an age- and gender-matched control population.^2^ A subsequent multicenter cohort study documented a 4% preoperative VTE rate for CS patients, and 3.4% of Cushing’s disease patients experienced VTE after transsphenoidal surgery (TSS) despite postoperative thromboprophylaxis with low-molecular-weight heparin. No VTE occurred among control patients undergoing TSS for nonfunctional pituitary adenomas.^3^

We present an unusual case of portal vein thrombosis (PVT) complicating CS caused by an adrenal adenoma. A PubMed search with the terms “portal vein thrombosis” and “Cushing’s syndrome” yielded only one brief French language case report^4^; to the best of our knowledge, this is only the second reported case of CS complicated by spontaneous PVT and the first English language case report.

## Case Presentation

**Figure 1.**
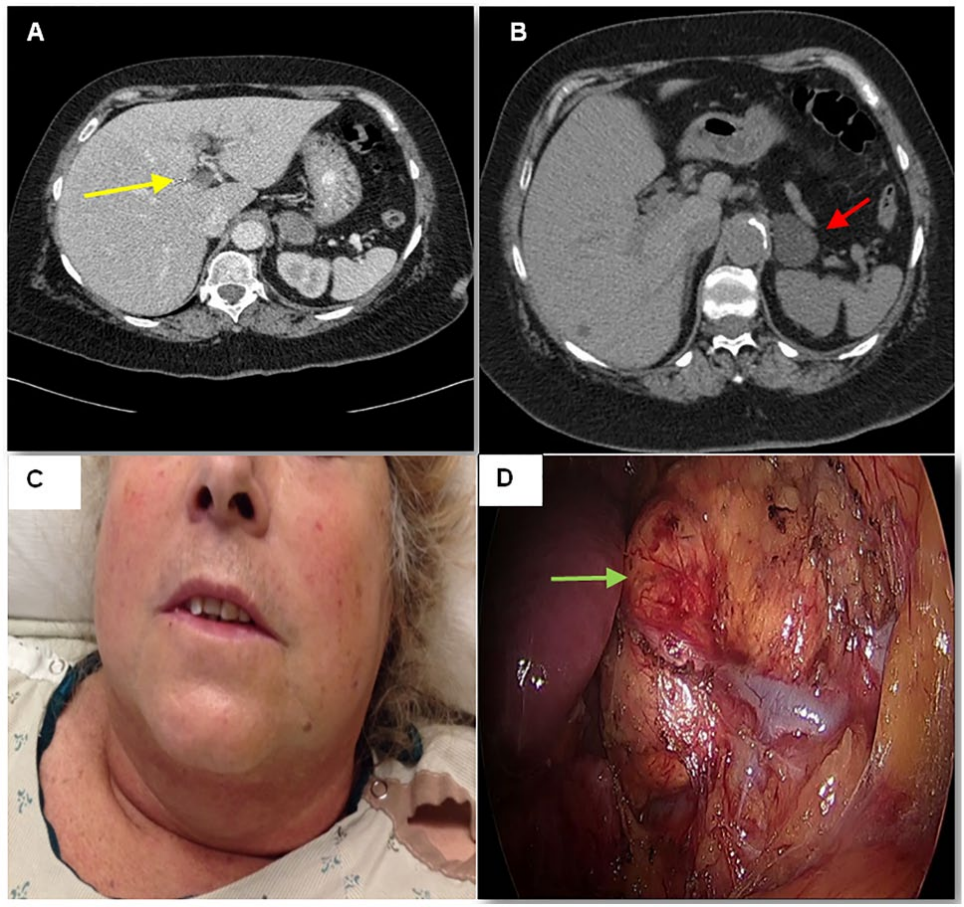
(A) Abdominal CT demonstrating left intrahepatic portal vein thrombosis (yellow arrow). (B) Noncontrast CT image of the left adrenal nodule revealing low attenuation and homogeneous appearance (red arrow). (C) Photograph of the patient’s head and neck demonstrating moon facies, plethora, and mild hirsutism. (D) In situ view of the left adrenal nodule prior to surgical resection (green arrow).

A 61-year-old female presented to the hospital for evaluation of right upper quadrant abdominal pain. The patient was febrile, and initial laboratories revealed leukocytosis (19,150/mm^3^, 4.00-10.80), mild elevation of alkaline phosphatase level (165 units/L, 50-130), and hypokalemia (2.9 mmol/L, 3.5-5.0). Neither cholescintigraphy nor right upper quadrant abdominal ultrasound showed evidence of cholecystitis, but the left portal vein was found to be occluded by ultrasonography. No sonographic features of cirrhosis (eg, increased echogenicity, significant nodularity, or right hepatic lobe atrophy) were observed. Left PVT was subsequently confirmed by computed tomography (CT; Figure 1A). Abdominal CT also revealed an incidental 3.8 × 3.2 cm left adrenal mass with radiographic features favoring adenoma including regular borders and homogeneous, low-density attenuation (−9 Houndsfield units) on noncontrast images (Figure 1B). No lower extremity venous thrombi were detected by ultrasound venous Doppler imaging.

Endocrinology consultation was obtained due to discovery of the left adrenal mass. CS was suspected due to truncal obesity, prominent dorsal cervical fat pad, moon facies, plethora, and hirsutism on examination (Figure 1C) and hypokalemia at initial presentation. Hypercortisolemia was confirmed by multiple diagnostic tests including overnight dexamethasone suppression test, midnight plasma cortisol, midnight salivary cortisol, and 24-hour urine free cortisol (Table 1). Morning ACTH level was <10 pg/mL (Table 1), indicating ACTH-independent hypercortisolism.

**Table 1.** Biochemical Evaluation for Cushing’s Syndrome.

Parameter	Result	Reference Range
Midnight plasma cortisol (µg/dL)	10.5	<7.5
Midnight salivary cortisol (µg/dL)	550	<100
24-Hour urine free cortisol (µg)	146	3.5-45
8 am ACTH (pg/mL)	7.2	10-60

The patient also underwent a simultaneous hypercoagulability workup. Evaluation for procoagulant antibodies (eg, antiphospholipid antibodies); deficiencies of protein C, protein S, and antithrombin III; prothrombin G2021A mutation; and activated protein C resistance was unremarkable. Factor VIII activity was significantly elevated at 365% (50% to 149%).

PVT due to hypercoagulability caused by a cortisol hypersecreting left adrenal adenoma was diagnosed. Fever was felt to be a sign of pylephlebitis, so the patient was managed acutely with unfractionated heparin and the combination vancomycin, levofloxacin, and metronidazole. Fever and abdominal pain resolved quickly with anticoagulation and antimicrobial therapy. Warfarin was titrated to therapeutic international normalized ratio, and the patient was discharged home to complete a 3-month course of oral anticoagulation. Laparoscopic left adrenalectomy was subsequently performed (Figure 1D), with glucocorticoids started for empiric treatment of secondary adrenal insufficiency. Postoperative histopathology confirmed an adrenal adenoma. Hydrocortisone was tapered off over 6 weeks following surgery, and the patient resumed warfarin for a total of 6 months oral anticoagulation. The patient’s postoperative course was uneventful.

## Discussion

Increased risk of VTE in CS appears to be due cortisol-induced changes to all 3 components of Virchow’s triad—vascular endothelial injury, hypercoagulable state, and venous stasis.^5^ Several humoral markers of endothelial dysfunction, including endothelin-1,^6^ homocysteine,^7^ osteoprotegerin,^8^ and vascular endothelial growth factor^9^ are elevated in CS, and endothelial dependent vasodilatation is also measurably impaired.^10^ Multiple coagulation factors are increased in CS, particularly factor VIII and von Willebrand factor (vWF).^11^ Glucocorticoid excess also increases plasminogen activator inhibitor type 1 (PAI-1) levels and activity.^12^ The combination of increased coagulation factors and decreased fibrinolytic capacity predisposes to venous thrombosis. Neither vWF nor PAI-1 was measured in this case, but factor VIII level was significantly elevated (365%). Levels above 160% are associated with a 4-fold increased risk of VTE.^11^ Some CS patients develop a secondary polycythemia that may cause venous stasis from hyperviscosity, and decreased activity, especially immobilization after surgery, also predisposes to venous stasis and thrombosis.^11^

Our patient’s case is unusual because most cases of CS-associated VTE occur in the lower extremities and PVT is rare. In a large series of 307 patients with CS, 29 (9.3%) experienced VTE, and the vast majority of patients (27/29, 93%) were diagnosed with deep vein thrombosis (DVT).^13^ Ten of the 27 patients with DVT also experienced fatal or nonfatal pulmonary embolism (PE), and another patient experienced a fatal PE without reported DVT. A recently published review of 13 patient series and over 1300 cases of CS found a similar rate of VTE (8.9%), with almost all patients experiencing lower extremity DVT and/or PE.^14^ Slightly more cases of VTE occurred after surgery rather than spontaneously.

An autopsy series from Malmö, Sweden, with nearly 24 000 patients found a 1% cumulative incidence of PVT.^15^ Secondary hepatobiliary malignancy, cirrhosis, primary hepatobiliary malignancy, abdominal infections or inflammatory conditions, and myeloproliferative disorders were the major etiologies of PVT in descending order of frequency and collectively accounted for 86% of PVT cases. No cases of PVT associated with CS were reported in the Swedish autopsy series, and PubMed searches linking the terms “Cushing’s syndrome,” “hypercortisolemia,” “hypercortisolism,” “cortisol,” and “portal vein thrombosis” yielded only the single French language case report from Tunisia previously noted. There appear to be at least 2 key reasons why CS is less likely to cause thrombosis in the portal venous circulation than the lower extremity venous circulation: (1) hypercortisolemia does not cause the thrombophilia states—factor V Leiden mutation, protein C deficiency, protein S deficiency, antithrombin deficiency, and antiphospholipid antibodies—that are most commonly associated with PVT^16^ and (2) blood flow rate through the portal venous circulation measured by duplex ultrasonography (DUS)^17^ is 2- to 20-fold higher than DUS measured lower extremity venous blood flow depending on the vein evaluated.^18^

Perioperative thromboprophylaxis appears to reduce the risk of postoperative VTE for CS patients, though relatively little is published on the topic and there are no clear recommendations regarding the type or duration of anticoagulation. Boscaro and colleagues^13^ compared the rate of VTE in 75 patients who did not receive postoperative anticoagulation (group 1) to 232 patients who received unfractionated heparin for at least 2 weeks followed by warfarin for at least 4 months (group 2). VTE rate was reduced from 20% in group 1 to 6% in group 2, and survival analysis demonstrated a significantly lower rate of morbidity and mortality from thromboembolic events in group 2. Recently, Barbot and colleagues^19^ compared 2 groups of Cushing’s disease patients undergoing TSS; group A received fractionated heparin from postoperative day 1 to discharge or a total of 14 days plus glucocorticoid replacement, and group B was managed with 30 days of fractionated heparin, compression stockings, early ambulation, and omission of glucocorticoids. There were no VTE events in group B compared to 3 in group A, though the difference was not quite statistically significant. The hypercoagulable state of CS may persist well beyond the time that cortisol levels return to normal. Markers of hemostasis and fibrinolysis failed to change significantly after 80 days in a group of patients with Cushing’s disease managed medically to biochemical remission of hypercortisolemia as indicated by normalization of 24-hour urine-free cortisol.^20^ Though PVT was our patient’s initial thromboembolic event, she was managed with a 6-month course of warfarin due to occurrence of VTE in the setting of CS.

Elevated serum cortisol induces changes in hemostatic and fibrinolytic pathways that create a prothombotic state, and this manifests clinically as increased risks of spontaneous and postoperative VTE. Lower extremity DVT and PE account for the significant majority of thrombotic events in CS patients. We present a case of CS due to an adrenal adenoma as the etiology of PVT, a rare clinical event that mostly occurs as a complication of cirrhosis or hepatobiliary malignancy. This appears to be only the second reported case of PVT complicating CS and the first in the English language peer-reviewed literature. The patient responded well to initial treatment with unfractionated heparin and antimicrobial therapy followed by laparoscopic adrenalectomy and a 6-month course of warfarin. Prolonged anticoagulation for patients with CS and VTE appears advisable given potential persistence of a hypercoagulable state beyond initial resolution of hypercortisolemia. Though lower extremity DVT and PE are the most likely complications of hypercoagulability in CS, this case illustrates that CS also has the potential to cause PVT.
